# Learning navigation – Learning with navigation. A review

**DOI:** 10.1051/sicotj/2017025

**Published:** 2017-06-02

**Authors:** Jean-Yves Jenny, Frederic Picard

**Affiliations:** 1 Centre for Orthopedic and Hand Surgery (CCOM), University Hospital of Strasbourg 10 Avenue Baumann 67400 Illkirch France; 2 Orthopaedic Department Golden Jubilee National Hospital and Biomedical Engineering Strathclyde University Agamemnon Street G81 4DY Glasgow UK

**Keywords:** Navigation, Learning, Teaching, Review

## Abstract

The goal of this review paper is to retrieve from the existing literature relevant information (1) about the learning curve of the currently existing navigation systems and (2) about the use of navigation system for teaching orthopaedic procedures. All studies reporting on the learning curve of navigation systems support the hypothesis that computer-navigated total knee arthroplasty (TKA) involves only a short learning curve and that beginners can obtain good results from the beginning of their experience, as navigation provides continuous feedback during all phases of the knee replacement surgery and allows for correcting any bone cut errors. Interestingly, there is no comparable research on the learning curve of TKA with standard, manual instrumentation. One might postulate that this learning curve might be longer than with navigation, with potentially a higher rate of outliers. The current literature does support that navigation may be an efficient teaching tool for both experienced orthopaedic surgeons and trainees. Experienced surgeons may improve their skills with conventional techniques and learn new techniques more efficiently and more quickly. Trainees may have a better understanding of the procedure and learn standard techniques with a shorter learning curve. This is probably due to the immediate feedback of navigation systems. A shorter learning curve may be associated with improved clinical and functional results for the patient during this critical period. However, there is no evidence that training with navigation excludes trainees from the need to work in academic environments with experienced teachers. Future techniques in training may include the development of laboratory simulation procedures using navigated feedback.

## Introduction

The impact of limb and implant alignments after total knee arthroplasty (TKA) remains debated [1]. However, it is generally accepted that excessive tibial varus or tibial and femoral malrotation may negatively influence clinical outcome [2].

Computer-assisted surgery (CAS) was developed to improve the accuracy of surgical procedures. It has been extensively demonstrated that the use of CAS is associated with a better accuracy and better precision when performing hip or knee arthroplasties [3, 4]. Unlike robotic surgery, which aims to replace the surgeon’s work by a guided device [5], navigation keeps the surgeon himself involved in the procedure [6]. Navigation systems do not take any decisions during the surgical procedure, but help the surgeon by providing more reliable information in order to take appropriate action themselves. The registration process is performed by the surgeon first, and then the computer analyses the data and provides the surgeon with the relevant information to place the implant in the expected position. This registration process may lead to inaccurate results if it is not done properly (“garbage in, garbage out”).

Introducing a new technique during a surgical procedure involves a learning curve [7] and should be followed by evaluation of the technical quality of this change. Methods for quality control have been initially developed for industrial quality control and adapted to medical research [8, 9]. Sequential outcome measures are considered measurements of a process. It is generally accepted that the use of navigation systems during orthopaedic procedures requires significant skills [10]. Therefore, it may involve a significant learning curve, but it is unclear if such systems should be released to experienced orthopaedic surgeons only [10, 11]. The immediate feedback generated by the use of navigation during orthopaedic procedures may facilitate resident teaching when they assist experts, and may help junior surgeons to avoid implant malposition during their learning curve of hip or knee replacement [10]. The goal of this review paper is to retrieve from the existing literature relevant information (1) about the learning curve of the currently existing navigation systems and (2) about the use of navigation system for teaching orthopaedic procedures.

## Learning curve of navigation systems

Any new surgical procedure involves a learning curve until the steady state is obtained. Common sense dictates that the more sophisticated the procedure is, the longer the learning curve will be. Navigation systems are generally considered by the non-user surgeons as sophisticated and tough procedures, especially because computer use is still uncommon in the operative field. However, learning curves of different navigation systems have been assessed, specifically for total knee arthroplasty (TKA).

Nizard et al. [12] analysed the learning curve after the introduction of a computer tomography based navigation system in one single institution (Navitrack^®^ [13]). They used an industrial tool, the cumulative sum test (CST), which allows a sequential quality process control and is well adapted to get an early feedback about surgical performance [14, 15]. This test may determine if the process is in control, and if some characteristics of the process (such as the mean) deviate from a pre-specified target value. The authors recorded 78 TKAs in 72 patients. The main outcome studied was overall alignment measured on long leg weight-bearing radiographs taken two to 12 weeks after surgery. Control of the process was obtained during the entire study, and the learning curve was completed after 27 TKAs. The CST showed that, for the first 19 knees treated, a constant systematic varus deviation was observed, followed by eight knees with symmetric valgus deviations. After these 27 knees, the process seemed to stabilize at the 180° target, with a slight trend towards varus knees. Sporadic out-of-control implantation was observed for two femur components and four tibia components. Operating time was considered under control during the whole study. The outlier rate (more than 3° off the neutral coronal alignment) was 10%. Seventeen percent of the femur components and 25% of the tibia components had a deviation of more than 2° off the neutral coronal alignment.

Jenny et al. [16] analysed the learning curve after the introduction of a non-image based navigation system in a multicentre study (OrthoPilot^®^ [17]). They compared the results of implantation of the same TKA with the same navigation system in five centres experienced with navigation use to eight centres with no navigation experience. The main outcome of the study was overall alignment measured on long leg weight-bearing radiographs taken three months after surgery. The study included 368 cases: 150 in the control group and 218 in the study group. No difference was observed for the radiological accuracy of implantation between experienced and beginner centres. In the experienced centres, 136 cases (91%) were within the desired range of ±3° while 191 cases (88%) in the beginner centres were (*p* > 0.05). There was no difference for the accuracy of implantation of both femur and tibia components. There was no difference in the clinical evaluation or occurrence of complications between both groups. The only observed difference was in the mean operating time: 107 ± 26 min in the experienced group vs. 118 ± 23 min in the beginner group (*p* < 0.001). After 30 knee replacements, the two logarithmic regression curves of the study and the control groups showed to be nearly parallel at study completion. There was no significant variation in operating time in the control group, whereas there was a tendency of decreased operating time in the study group with growing surgical experience. The authors concluded that there was no learning curve for accuracy of implantation. The only learning curve was about operating time, and was completed after 30 cases.

Smith et al. [18] performed a prospective study comparing the first 50 computer-navigated primary TKAs performed by a single consultant with no previous experience of navigation but significant experience in non-navigated primary TKAs to 50 computer-navigated primary TKAs performed by an expert navigator during the same period. The same OrthoPilot^®^ non-image based navigation system as in the Jenny et al. [16] study was used. Outcome measurements were Oxford knee score and coronal mechanical femoro-tibial angle measured on a long leg weight-bearing digital hip-knee-ankle radiographs at six weeks and one year after surgery. There was a significant 19-min increase in the operating time for the novice surgeon during the first 20 cases, but this difference disappeared during the next 20 cases. There was no difference between the two surgeons for other clinical or radiographic data. The authors concluded that a surgeon novice in navigation was able to achieve the clinical and radiographic results of an expert from the beginning of his experience with navigation. As in the Jenny et al. [16] study, the only significant difference was an increased operating time, which decreased quickly, with no remaining difference after 20 cases.

Confalonieri et al. [11] performed a prospective study comparing three groups each with 25 patients undergoing computer-assisted TKA who were operated on by a surgeon experienced in both TKA and navigation, a surgeon experienced in TKA but with no experience in navigation, and a general orthopaedic surgeon with a low volume TKA experience and no experience in navigation. A non-image based navigation system was used (VectorVision^®^ [19]). The deviation from the expected cut orientation and the number of recuts (if the deviation was superior to three degrees) were recorded. No significant differences were seen for the distal femoral cut and the proximal tibia cut in the coronal plane. A statistically significant inferior result was observed for the general orthopaedic surgeon for the distal femoral cut and the proximal tibia cut in the sagittal plane. There was a negative correlation between the level of experience both in computer navigation and knee replacement surgery and the number of recuts. There was no statistically significant difference between the three surgeons for all radiographic parameters. There was a negative correlation between the level of experience both in computer navigation and knee replacement surgery and the operating time. The authors concluded that computer navigation was a useful tool to train inexperienced surgeons in TKA.

Sampath et al. [20] investigated the relationships between the tourniquet time of a single surgeon and severity of the preoperative deformity, body mass index and surgeon’s experience measured by the number of navigated TKAs performed by the surgeon prior to the considered procedure. One hundred and seventy-two cases of navigated TKAs were analysed. The OrthoPilot^®^ navigation system was used. A statistically significant relationship was observed between tourniquet time and the total number of previous TKAs performed, with a 0.1 min decrease for each additional TKA, suggesting that the learning curve might last longer than previously assessed. No outliers were observed when considering the radiographic prosthetic orientation.

Shields et al. [21] analysed the learning curve of a single surgeon with previous vast experience in resurfacing hip arthroplasty (RHA). They performed a match-paired, retrospective comparative study comparing conventional procedures to navigated procedures during the introduction period of this new procedure. There was no difference in the mean orientation of the implants in the two groups. There was no difference in the operating time in the two groups. The authors concluded that there was no significant learning curve for this specific, experienced hip surgeon when introducing navigation for RHA.

All these studies have similar conclusions. The non-expert surgeons are immediately able to take advantage of the navigation technique when considering the accuracy of bone cuts, whatever the navigation system used. However, these surgeons generally need more operating time than experts, and this learning period may last 20 to 30 TKAs. The duration of this learning curve did not appear significantly impacted by the system used. No information is currently available for the non-TKA procedure.

No study specifically addressed the reasons for this prolonged operating time. One may speculate that the use of new instruments, a new surgical workflow or familiarization with the computer interface may significantly impact the speed of the process. The immediate feedback about the component position may avoid bone recuts, but rapid placement of the resection guides in the optimal 3D orientation is not intuitive. All these points could be addressed in simulated laboratory conditions, which may decrease the learning curve period further.

### Conclusion

All studies support the hypothesis that computer-navigated TKA involves only a short learning curve and that beginners can obtain good results from the beginning of their experience, as navigation provides continuous feedback during all phases of the knee replacement surgery and allows for correcting any bone cut errors. Interestingly, there is no comparable research on the learning curve of TKA with standard, manual instrumentation. One might postulate that this learning curve might be longer than with navigation, with potentially a higher rate of outliers.

## Teaching with navigation

### Can experienced surgeon improve their skills with navigation?

It has been extensively proven that performing a TKA with the help of a navigation system allows positioning the implants in a more accurate and more reproducible position than when a conventional instrumentation is used [3, 22]. Very few papers reported no difference between navigated and conventional implantation [23, 24]. Similar results have been published for unicompartmental knee arthroplasty, which is known to be technically more demanding than the TKA [25–27]. Similar results have also been published for acetabular and femoral components of total hip arthroplasty [4, 28]. Most of these studies were performed by experienced surgeons in high volume centres. It can be concluded that even experienced surgeons may improve their skill while implanting joint replacement under navigation control. However, the question of the clinical relevance of this improvement is still debated, with conflicting positive [29, 30] or negative [31, 32] impacts of navigation.

Besides improving the accuracy of bone cuts orientation, there is some evidence that navigation may help experienced surgeons improve their skills in balancing soft tissue, especially during the TKA [33–36].

Navigation may also help experienced surgeons even when they use the conventional implantation technique for TKA [37].

Iorio et al. [38] analysed 150 TKAs operated by the same, high volume knee surgeon. Three different periods of time were considered: first, patients operated with a conventional technique before any computer-assisted experience; second, patients operated with computer-assisted surgery; finally, patients operated with a conventional technique after having gained experience in computer-navigated techniques. The outcome was evaluated by measuring the coronal limb alignment on long leg weight-bearing radiographs. Respective rates of optimal placement were 68% in the first period, 92% in the second period and 82% in the final period. The difference was significant between first and second periods as well as between first and third periods, but not between second and final periods. Navigation had an educational role and helped improve the ability of the surgeon in positioning prosthetic components more precisely in the coronal plane even when navigation was not used.

### Can experienced surgeon learn a new technique with navigation?

Romanowski and Swank [39] reported a single surgeon series of 71 consecutive RHA in which the components were placed with the use of computer-assisted navigation. Orientation of the implants and operative time were compared in three sequential operative time periods including the introduction period of this new procedure. Over three sequential operative time periods, computer-assisted navigation produced consistent values with regard to intraoperative cup inclination (43°, 44° and 40°) and postoperative radiographic alignment of the cup (46°, 44° and 43°) and femoral stem (148°, 147° and 144°), despite different levels of surgeon experience. Operative times significantly decreased with surgeon experience, showing the largest decrease after the first and second sequence intervals (110, 98 and 95 min, respectively). There was a significant difference with evolving surgeon experience concerning intraoperative stem placement (144°, 142° and 138°, respectively) despite the mean values remaining well clustered. No femoral notching occurred throughout the series. The authors concluded that computer-assisted navigation was a reliable technique for accurate placement of RHA. Furthermore, computer navigation allowed for consistency of component alignment independent of surgeon’s experience.

Shields et al. [21], in the previously quoted paper, analysed component placement in RHA. Eighty-nine percent of the femoral components were placed in the expected valgus position in the navigation group, compared with only 80% in the conventional group, with a narrower range of variation from the target in the navigated group. The authors concluded that this experienced surgeon had been able to improve his skill in RHA by using navigation assistance.

### Can trainees learn a new technique with navigation?

Motor learning theory suggests that, while the real-time feedback provided by computer-assisted orthopaedic surgery should improve performance, it may be detrimental to learning. Gofton et al. [40] analysed the impact of computer-assisted technology on trainee performance during total hip arthroplasty (THA). Fifty-five trainees were randomized to one of the three training groups: conventional training, computer navigation or conventional implantation with navigation control. Outcomes were assessed in a pretest session and in 10-min and six-week retention and transfer tests. All groups demonstrated improved accuracy and precision in cup placement during training. The computer navigation group demonstrated significantly better accuracy and precision in early training and better precision throughout training. No significant degradation in performance was observed between the immediate and the delayed testing for any group, suggesting that there was no detrimental effect of any of the tested training modalities on learning.

Cobb et al. [41] analysed the influence of navigation on the learning curve and accuracy of RHA by trainees. Twenty trainees participated in a randomized trial. Instruction was given to all participants about the surgical technique of RHA, the use of conventional instrumentation, the use of a computed tomography-based planning and the use of a navigation system. The trainees were then split into three groups undertaking these tasks in three different orders. The mean error to insert the guidewire was 23° with the conventional instrumentation, 22° with the computed tomography (CT) plan technique and 7° using navigation. Trainees produced similar accuracy, even in their first attempt, on difficult anatomy when using navigation. Motivated students rapidly achieved an expert level of accuracy when provided with navigation. Learning a conventional method first did not improve performance, even in difficult cases. The authors suggest that navigation may play an important role in reducing the learning curve of trainees in hip resurfacing arthroplasty.

Nousiainen et al. [42] determined the impact of a computer-navigated training model on the learning curve of trainees in the fixation of femoral neck fractures. They conducted a multicentre, prospective, randomized, controlled study with surgical trainees with no prior experience in this procedure. After a training session, participants underwent a pretest by performing the screw placement on a simulated hip fracture using fluoroscopic guidance. Immediately after, participants were randomized into either undergoing a training session using conventional fluoroscopy or computer-based navigation. Immediate post-test, retention (four weeks later), and transfer tests were performed. Performance during the tests was determined by radiographic analysis of hardware placement. Screw placement by trainees was ultimately equal to the level of an expert surgeon with either training technique. Participants who trained with computer navigation took fewer attempts to position hardware and used less fluoroscopy time than those trained with conventional fluoroscopy. When those trained with conventional fluoroscopy used computer navigation at the transfer test, less fluoroscopy time and dosage were used. The authors suggested that computer navigation may be safely used to train surgical novices in a basic procedure, avoiding training on a real patient.

Khakha et al. [43] compared the mid-term clinical outcome of TKA performed by a consultant orthopaedic surgeon to that of trainees. Ninety-two patients were matched and randomly allocated to have CAS performed by either a consultant or a trainee and followed up prospectively for five years. The data demonstrated that trainees were able to achieve equal coronal alignment, blood loss and functional scores. However, consultant surgeons had a significantly shorter tourniquet time. The authors concluded that CAS can assist less experienced surgeons to reliably achieve good mid-term outcomes in TKA.

Love and Kinninmonth [44] analysed the effectiveness of computer navigation as a training tool in TKA. They performed a training exercise on plastic models to simulate cutting guide movement due to inadequate fixation, the effect of using slotted or open cutting guides, the effect of bending the saw blade and the effect of recutting on the accuracy of the targeted resection. The immediate feedback provided by computer-navigated TKA allowed both surgeons and trainees to immediately identify errors in surgical technique and to correct them precisely.

Schnurr et al. [45] analysed whether trainees may implant knee prostheses using computer navigation as accurately as experienced consultants. Six hundred and sixty-two consecutive TKAs performed by consultants or trainees under supervision by a consultant were analysed retrospectively. Operation time was significantly longer for trainees vs. consultants (139 vs. 122 min, respectively). There was no significant difference between trainees and consultants for cutting errors. The rate of outliers with a mechanical axis deviation >2° was low and not significantly different between trainees and consultants. The authors concluded that trainees may implant their first TKA using computer navigation as accurately as experienced consultants at the expense of a longer operative time.

### Conclusion

The current literature does support that navigation may be an efficient teaching tool for both experienced orthopaedic surgeons and trainees. Experienced surgeons may improve their skills with conventional techniques and learn new techniques more efficiently and more quickly. Trainees may have a better understanding of the procedure and learn standard techniques with a shorter learning curve. This is probably due to the immediate feedback of navigation systems. A shorter learning curve may be associated with improved clinical and functional results for the patient during this critical period. However, there is no evidence that training with navigation exempts trainees from the need to work in academic environment with experienced teachers. Future techniques of training may include the development of laboratory simulation procedures using navigated feedback.

## Conflict of interest

JYJ receives royalties from Aesculap, is a paid consultant for Exactech and FH Orthopedics.

FP has patents and licenses with Brainlab via CMU, BBraun and Oxford University Press. He received financial support for symposia from Stryker, BBraun, Convatec and Blue Belt Technology (BBT). His research institution received research funding from Stryker, BBraun, Bayer, Mathys, Zimmer, Convatec, BBT.
